# Correlation of handgrip strength with quality of life-adjusted pulmonary function in adults

**DOI:** 10.1371/journal.pone.0300295

**Published:** 2024-03-11

**Authors:** Hae In Jung, Kang-Mo Gu, So Young Park, Moon Seong Baek, Won Young Kim, Jae-Chol Choi, Jong-Wook Shin, Jae-Yeol Kim, Young D. Chang, Jae-Woo Jung

**Affiliations:** 1 Division of Pulmonary and Allergy Medicine, Department of Internal Medicine, Chung-Ang University Hospital, Seoul, Korea; 2 Division of Pulmonary and Allergy Medicine, Department of Internal Medicine, Chung-Ang University College of Medicine, Seoul, Korea; 3 Department of Supportive Care Medicine, Moffitt Cancer Center, United States of America; 4 Department of Oncologic Sciences, University of South Florida, Tampa, Florida, United States of America; Hamasaki Clinic, JAPAN

## Abstract

**Background:**

Handgrip strength (HGS) is acknowledged as a key indicator of overall physical fitness and is associated with various health outcomes.

**Objectives:**

This research investigates the correlation between HGS and quality of life (QoL), focusing on its relation to pulmonary function in the general adult population.

**Method:**

The study involved 19,402 participants aged 40 and above, spanning from 2014 to 2019, who underwent pulmonary function and HGS tests. Participants were categorized based on lung function, and regression analyses were employed to examine the relationship between HGS and QoL, with adjustments made for lung function.

**Results:**

The average age of the cohort was 58.2 years, comprising 44.6% males and 41.2% smokers. Out of the 18,708 participants who completed the European Quality of Life Scale-Five Dimensions (EQ-5D-3L) assessment, higher severity levels in mobility, self-care, usual activities, pain or discomfort, and anxiety or depression were linked to lower HGS in both sexes. Additionally, among the 3,723 participants who completed the Health-related Quality of Life Instrument with 8 Items (HINT-8) assessment, higher severity levels in pain, work, and depression were associated with lower HGS in men. In women, higher severity levels in climbing stairs, pain, vitality, and work correlated with lower HGS.

**Conclusions:**

As problems indicated by EQ-5D worsened, there was a consistent decrease in handgrip strength (HGS) across both genders. The HINT-8 assessment further revealed that increased severity in pain and work-related issues led to reduced HGS in both men and women. This study highlights the relationship between HGS and Quality of Life (QoL), taking lung function into consideration, and underscores the importance of HGS as a potential marker of physical health and fitness.

## Introduction

Handgrip strength (HGS) is widely recognized as a simple and reliable measure for assessing muscle strength [1]. It has gained attention for its potential to provide insights into overall health and is acknowledged as an effective tool for monitoring lung function in diverse populations [2]. A meta-analysis of 25 studies indicated weak to moderate associations between HGS and lung function in 87% of healthy adults [3].

Quality of Life (QoL) encompasses overall well-being and satisfaction across various life dimensions. Health-Related Quality of Life (HRQoL), a subset of QoL, focuses on health status and reflects functional limitations, symptoms, and psychological effects stemming from an individual’s health condition [4].

Previous studies have investigated the correlation between muscle strength, particularly HGS, and HRQoL. A 2018 study in Korea found an association between lower HGS and reduced QoL in terms of mobility and pain/discomfort, as measured by the EQ-5D assessment [5]. Similarly, a 2020 study in rural China showed a significant correlation between HGS and both EQ-5D index and VAS scores in older adults [6]. These studies have provided foundational insights into how HGS is associated with overall health satisfaction.

This study aims to explore the relationship between HGS and HRQoL indicators in adults aged 40 years and older, categorizing pulmonary function using data from the Korea National Health and Nutrition Examination Survey (KNHANES) conducted between 2014 and 2019.

## Materials and methods

### Data collection and study participants

The Korea National Health and Nutrition Examination Survey (KNHANES) is a comprehensive survey conducted in South Korea to gather extensive data on health status, behaviors, and nutrition intake. Its aim is to provide nationally representative and reliable statistics, achieved through the collection of data from a representative sample of individuals [7]. The survey forms the basis for developing health policies in the country. The data analyzed in this study were collected between 2014 and 2019.

### Ethical approval and informed consent

The KNHANES received approval from the Institutional Review Board (IRB) of the Korea National Institutes of Health for the years 2014, 2018, and 2019 (IRB Nos: 2013-12EXP-03-5C, 2018-01-03-P-A, 2018-01-03-C-A). During 2015–2017, the survey was exempt from IRB review, as it was state-conducted research for public welfare as defined by the Bioethics Act and its Implementing Regulations. All participants provided informed consent. This study also received prior approval from the IRB of Chung-Ang University Hospital (IRB No.: 2302-008-19456).

### HGS measurements

HGS measurement was introduced in KNHANES in 2014. Participants underwent assessments using a digital grip strength dynamometer (T.K.K 5401, Japan). Initially, the highest value from three measurements of the dominant hand was recorded. Since 2018, this has been modified to record the maximum value from either hand.

### Definition of impaired pulmonary function

Pulmonary function tests (PFTs) were conducted on individuals aged 40 years and above, using calibrated spirometers in accordance with the 2005 ATS/ERS guidelines [8]. Initially, measurements employed a dry rolling seal spirometer, transitioning to a Vyntus Spiro in June 2016 [9]. Normalized predicted values for spirometry data were based on South Korea’s general population and obtained before bronchodilator use [10]. Normal lung function was defined as an FEV_1_/FVC ratio of ≥ 0.7 with an FVC of ≥ 80%. Obstructive lung function was characterized by an FEV_1_/FVC ratio < 0.7, while restrictive lung function was defined as an FEV_1_/FVC ratio of ≥ 0.7 with an FVC < 80%.

### QoL measurement (EQ-5D-3L)

EQ-5D, developed by the EuroQol group, is a standardized health status measure that offers a simple, generic assessment for clinical and economic purposes. Introduced in 1990, EQ-5D-3L consists of five questions evaluating different QoL aspects, including mobility, self-care, usual activities, pain/discomfort, and anxiety/depression [11]. Participants respond on a 3-point scale, with ‘1’ indicating no problems, ‘2’ some problems, and ‘3’ extreme problems. The EQ-5D index score, derived from a quality-weighted estimation study by the CDC, serves as a comprehensive health status measure [12].

### Health-related Quality-of-Life Instrument with 8 Items (HINT-8)

HINT-8, used in South Korea, assesses HRQoL across the general population [13]. It includes eight items covering physical health (climbing stairs, pain, vitality), social health (working), mental health (depression, memory, sleep), and a positive health domain (happiness). Participants rate each item on a four-point scale, from “no problems” to “mild,” “moderate,” and “severe problems.” Introduced in the 2019 KNHANES, HINT-8 is used alongside EQ-5D-3L for a more comprehensive assessment of HRQoL.

### Statistical analysis

Statistical analysis was performed using SPSS version 16.0. Continuous variables were presented as mean ± standard deviation (SD), and categorical variables as absolute numbers and percentages. Student’s t-test and one-way ANOVA were applied to continuous variables, while the χ2 test or Fisher’s exact test was used for categorical variables. Linear and multiple logistic regression analyses, adjusting for confounders such as age and pulmonary function, assessed the association between HGS and QoL. A significance level of p < 0.05 was considered statistically significant.

## Results

### Study population

**Fig 1:**
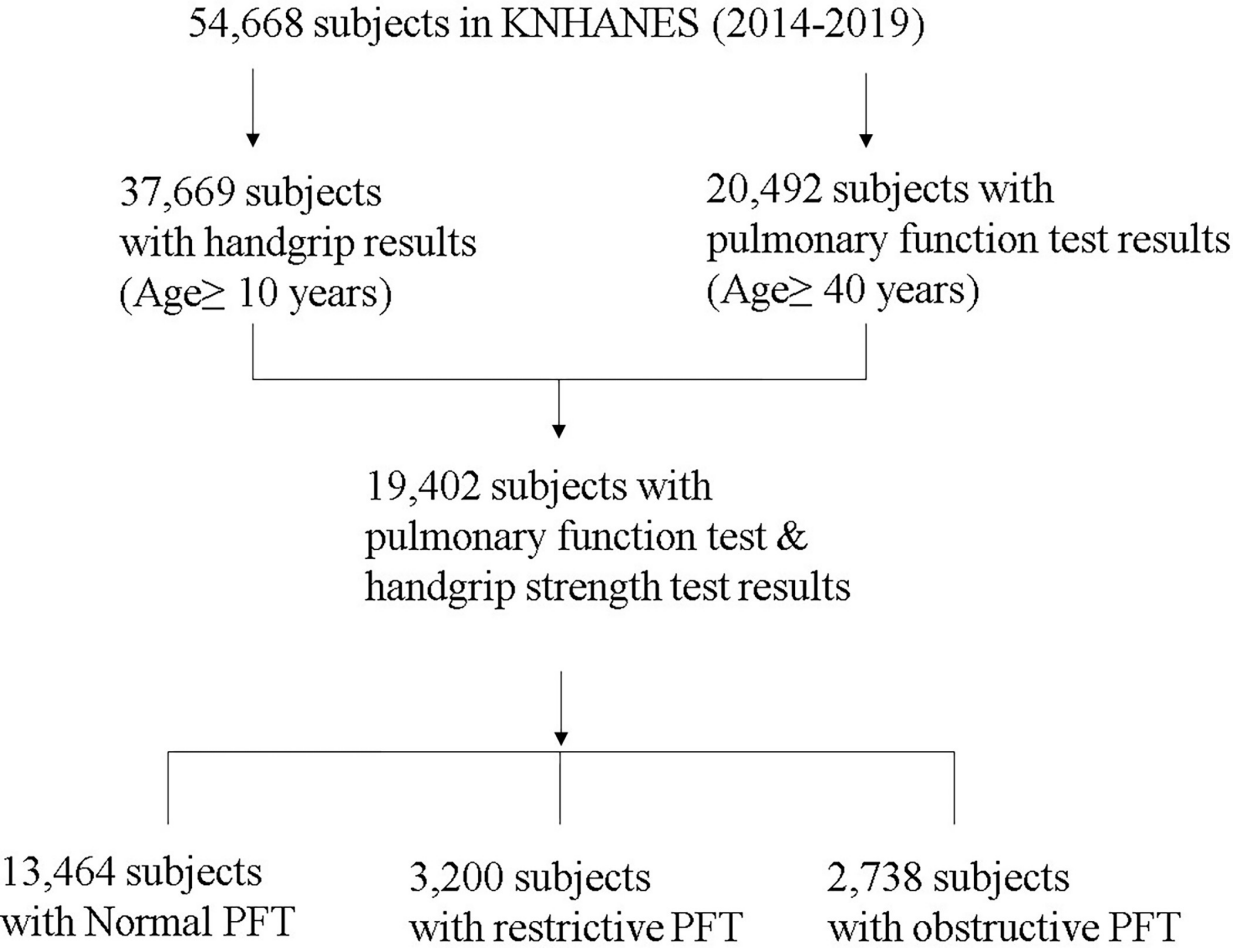
Flow of the study. Abbreviations: PFT: Pulmonary function test, KNHANES: Korean National Health and Nutrition Examination Survey.

Of the 54,668 individuals in the KNHANES data from 2014 to 2019, 20,492 participants underwent Pulmonary Function Tests (PFT), while 37,669 underwent Handgrip Strength (HGS) assessments. Among those aged above 40 years who completed both tests, 13,464 exhibited normal lung function, 3,200 had restrictive lung function, and 2,738 had obstructive lung function (Fig 1).

### Baseline characteristics

The mean age of the subjects was 58.2 ± 11.1 years, with males comprising 44.6% of the group. Among these participants, 41.2% were former or current smokers. The average Body Mass Index (BMI) was 24.2 ± 3.2 kg/m^2^. Comorbidities like hypertension and diabetes were present in 30.7% and 11.8% of the subjects, respectively. The average HGS for all subjects was 29.24 ± 9.95 kg. Subjects were categorized based on lung function into normal (69.4%), restrictive (16.5%), and obstructive (14.1%) groups. The mean HGS (± SD) for these groups were 29.1 kg (10.0) for normal, 27.9 kg (9.7) for restrictive, and 31.4 kg (9.6) for obstructive lung function, with significant differences observed across the groups (p <0.001). The prevalence of comorbidities and the EQ-5D index scores did not significantly differ among the three lung function groups (Table 1).

**Table 1:** Baseline characteristics of the study population.

Characteristic	Total	Normal	Restrictive	Obstructive	P-value*
	n = 19,402	n = 13,464 (69.4%)	n = 3,200 (16.5%)	n = 2,738 (14.1%)	
Age, years	58.2 ± 11.1	55.8 ± 10.5	61.4 ± 10.9	66.3 ± 9.7	
Male sex	8654 (44.6%)	5116 (38.0%)	1554 (48.6%)	1984 (72.5%)	
BMI, kg/m^2^	24.2 ± 3.2	24.0 ± 3.1	25.5 ± 3.6	23.8 ± 2.9	0.050
Smoking status					< 0.001
Never	11294 (58.9%)	8573 (64.3%)	1832 (57.8%)	889 (33.0%)	
Former smoker	4715 (24.6%)	2783 (20.9)	863 (27.25)	1069 (39.7%)	
Current smoker	3177 (16.6%)	1970 (14.8%)	473 (14.9%)	734 (27.3%)	
Comorbidities					
Hypertension	5872 (30.7%)	3355 (25.3%)	1351 (42.5%)	1166 (43.2%)	0.241
Diabetes mellitus	2256 (11.8%)	1209 (9.1%)	610 (19.2%)	437 (16.2%)	0.341
Chronic kidney disease	69 (0.4%)	31 (0.2%)	25 (0.8%)	13 (0.5%)	0.307
Liver cirrhosis	94 (0.5%)	56 (0.4%)	23 (0.7%)	15 (0.6%)	0.163
Pulmonary function test					
FVC, %	89.5 ± 12.6	93.9 ± 9.2	73.0 ± 6.5	87.2 ± 15.3	<0.001
FEV_1_, %	89.4 ± 13.7	94.9 ± 10.2	77.2 ± 8.2	76.5 ± 16.0	<0.001
FEV_1_/FVC, %	77.1 ± 7.5	79.3 ± 4.6	79.4 ± 5.0	63.5 ± 6.7	<0.001
Hand grip strength, kg	29.2 ± 9.9	29.1 ± 10.00	27.9 ± 9.7	31.4 ± 9.6	<0.001
EQ-5D index	0.94 ± 0.10	0.95 ± 0.10	0.93 ± 0.12	0.93 ± 0.12	0.083

Abbreviations: BMI: Body mass index, FVC: Forced vital capacity, FEV_1_: Forced expiratory volume at 1 second.

*P-value was adjusted for age and sex.

^£^EQ-5D; no problems (%), some problems (%), extreme problems (%).

### Association between HGS and EQ-5D

**Fig 2:**
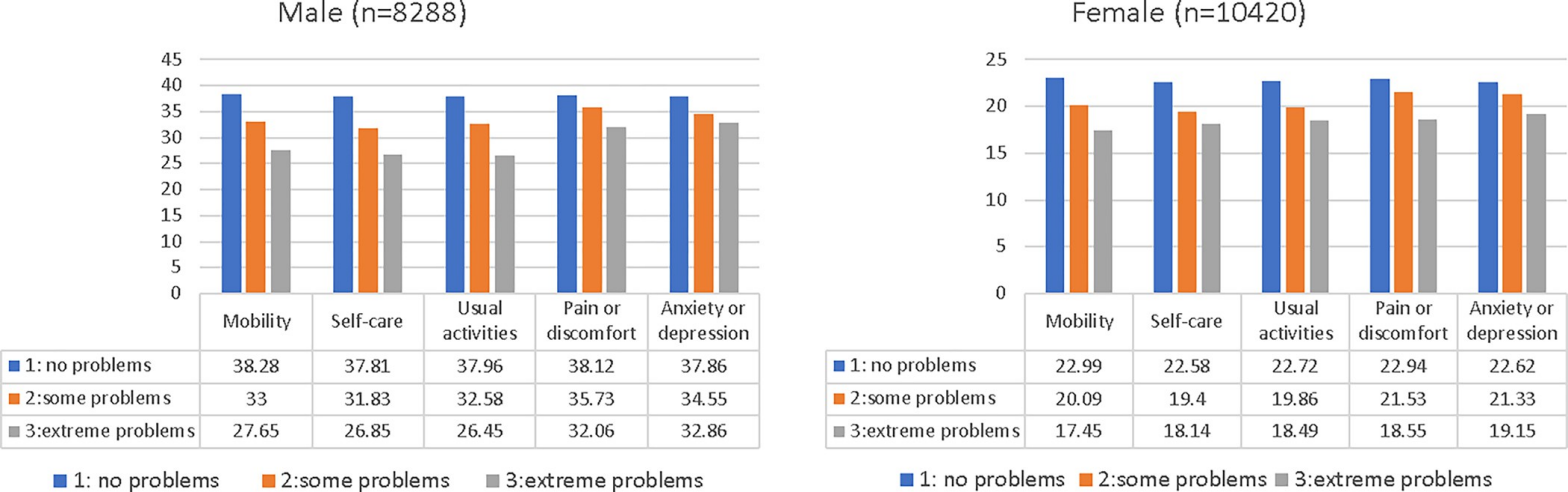
Association with handgrip strength and severity of European Quality of Life Scale-Five Dimensions (n = 18,708). Abbreviations: EQ-5D: European Quality of Life Scale-Five Dimensions. ^£^Level ‘1’: no problems, ‘2’: some problems, ‘3’: extreme problems. *P-value was adjusted by age and pulmonary function (normal, restrictive, and obstructive).

Among the 19,402 participants who underwent both PFT and HGS tests, 18,708 completed the EQ-5D (8,288 men and 10,420 women). Separate analyses for men and women were conducted to explore the relationship between HGS and EQ-5D, adjusting for age and categorized PFT groups. In both genders, lower HGS values were associated with higher severity levels in all five EQ-5D domains (mobility, self-care, usual activities, pain/discomfort, and anxiety/depression) (p < 0.001) (Fig 2, e-Table 1 in S1 File).

### Association between HGS and HINT-8

**Fig 3:**
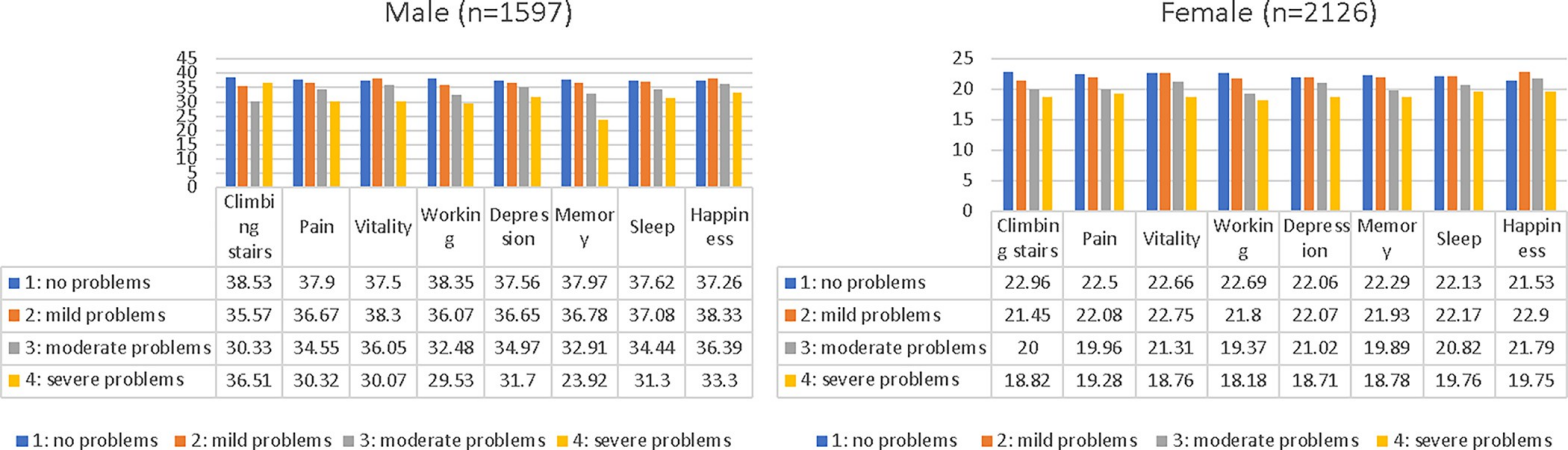
Association with handgrip strength and Health-related Quality of Life Instrument with 8 Items (n = 3723). ^£^Level ‘1’: no problems, level ‘2’: mild, level ‘3’: moderate, level ‘4’: severe problems. *P-value was adjusted by age and pulmonary function (normal, restrictive, and obstructive).

Of the 3,723 participants who completed the HINT-8 test in 2019, 1,597 were men and 2,126 were women. Variations in HGS were analyzed in relation to the severity levels of the eight health-related items. In men, higher severity levels in pain, working, and depression correlated with lower HGS. In women, increased severity levels in climbing stairs, pain, vitality, and working were associated with reduced HGS (Fig 3, e-Table 2 in S1 File).

## Discussion

This study, utilizing data from the KNHANES from 2014 to 2019, provides critical insights into the relationship between HGS and HRQoL, as well as lung function in adults aged over 40. A key finding is the consistent observation of lower HGS values in both men and women, correlating with increased severity in reported problems across various EQ-5D-3L domains. This trend was further echoed in the HINT-8 assessments, where elevated severity in pain and work-related issues corresponded with reduced HGS in both genders. Notably, in men, a severe depressive mood was also linked to lower HGS. These patterns highlight a potential connection between decreased HGS and poorer overall HRQoL. Importantly, this association persists even after accounting for the correlation between HGS and pulmonary function. These findings suggest a multifaceted role of HGS as an indicator, reflecting not only physical capacity but also broader health and well-being aspects in the adult population.

A previous study that utilized normative data showed that for the age group 55–59 years, HGS was 41.5±6.4 in men and 25.2±4.2 in women. For the 60–64 years age group, HGS was 39.7±6.3 in men and 24.2±4.1 in women [14] The differentiation in HGS cutoff values also points to the need for personalized health strategies. These strategies should not only address the physical decline associated with aging but also consider the varied impacts of diminished muscle strength on daily functioning and QOL.

Comparisons with previous studies, particularly those analyzing the relationships between EQ-5D-3L and HGS, have demonstrated consistent trends [5, 15]. This study observed that individuals reporting "no problems" across all EQ-5D domains exhibited significantly lower HGS, a trend persisting even after adjusting for age and pulmonary function—both recognized factors influencing HGS. The EQ-5D-3L, developed initially to assess quality of life (QoL) in health-vulnerable populations, is sensitive to sociocultural differences. However, it has limitations, such as a ceiling effect, where individuals frequently report no problems on all items, thereby reducing its effectiveness in distinguishing between various health conditions in healthy populations. To address these limitations, a Korean-specific tool, the HINT-8, along with its health utility calculation formula, was developed. These tools aim to comprehensively measure the HRQoL of the general Korean population and are used alongside EQ-5D-3L to provide a more multifaceted assessment of individual HRQoL. The introduction of the HINT-8 in 2019 offered additional insights into HRQoL for Koreans. Elevated severity levels related to pain, working, and depression were linked to lower HGS in men. Similarly, in women, associations were observed between reduced HGS and increased difficulty in climbing stairs, pain, vitality, and working. The study emphasizes the importance of incorporating HGS measurements when evaluating HRQoL indicators. These findings contribute to a more nuanced understanding of the interplay between HRQoL, HGS, and EQ-5D-3L, acknowledging the limitations of the latter and highlighting the complementary role of the HINT-8 in providing a comprehensive assessment of HRQoL.

The decline in muscle mass and strength associated with aging, particularly after the age of 40, serves as a significant biomarker for assessing health status and predicting future health outcomes, including mortality [14, 16–18]. The study establishes a noteworthy connection between decreased lung function, diminished muscle strength, and poorer QoL [5]. The findings suggest that incorporating interventions to maintain or enhance muscle strength could be a valuable strategy for promoting healthy aging and improving HRQoL.

It is important to acknowledge its limitations. First, the categorization of participants based solely on spirometry results may limit the ability to make specific disease diagnoses. This limitation is particularly relevant for conditions like Chronic Obstructive Pulmonary Disease (COPD), where the absence of post-bronchodilator (BDR) testing can affect the accuracy of diagnosis. Furthermore, the study’s cross-sectional design hinders the ability to establish causality in the observed relationships. This consideration is critical when interpreting the findings, as it suggests that the results indicate correlation rather than causation. Additionally, while the sample is representative of the South Korean population, the findings may not be directly applicable to populations in other regions with different demographic and health characteristics.

## Conclusion

This study highlights the strong association between HGS, lung function, and QoL in adults over 40. HGS emerges not just as a reliable indicator of muscle strength, but also as a valuable tool for identifying individuals who might benefit from targeted interventions aimed at improving strength and potentially predicting the risk of sarcopenia. The implications of these findings are far-reaching, emphasizing the importance of incorporating muscle strength assessment into routine health checks. Moreover, the relevance of HGS extends beyond mere physical health; it encompasses aspects of mental well-being as well. The strong correlation between HGS and various indicators of QoL highlights the necessity of integrating HGS assessments into clinical evaluations. Such integration would not only aid in a more comprehensive understanding of an individual’s overall health but also enhance their quality of life through more tailored health strategies and interventions.

## Supporting information

S1 File (DOCX)
