# Supplementary material for: Correlation of handgrip strength with quality of life-adjusted pulmonary function in adults
Source: PLoS One. 2024 Mar 11;19(3):e0300295. doi: 10.1371/journal.pone.0300295 (PMC10927077; doi:10.1371/journal.pone.0300295)
Supplement: S1 File — (DOCX) [file pone.0300295.s001.docx]

**e-Table 1. Association with handgrip strength and severity of European Quality of Life Scale-Five Dimensions (n = 18,708)**

1. Association with handgrip strength and EQ-5D-3L in male participants (n = 8288)

|  | 1 | 2 | 3 | Beta | P-value* |
| --- | --- | --- | --- | --- | --- |
| Mobility | 38.28 ± 7.56 | 33.00 ± 7.84 | 27.65 ± 5.99 | –0.119 | < 0.001 |
| Self-care | 37.81 ± 7.71 | 31.83 ± 7.99 | 26.85 ± 1.00 | –0.095 | < 0.001 |
| Usual activities | 37.96 ± 7.64 | 32.58 ± 8.29 | 26.45 ± 6.56 | –0.114 | < 0.001 |
| Pain or discomfort | 38.12 ± 7.63 | 35.73 ± 8.08 | 32.06 ± 7.88 | –0.092 | < 0.001 |
| Anxiety or depression | 37.86 ± 7.73 | 34.55 ± 8.02 | 32.86 ± 8.12 | –0.095 | < 0.001 |

Abbreviations: EQ-5D: European Quality of Life Scale-Five Dimensions.

^￡^Level ‘1’: no problems, ‘2’: some problems, ‘3’: extreme problems.

*P-value was adjusted by age and pulmonary function (normal, restrictive, and obstructive).

1. Association with handgrip strength and EQ-5D-3Lin female participants (n = 10,420)

| Level^￡^ | 1 | 2 | 3 | Beta | P-value* |
| --- | --- | --- | --- | --- | --- |
| Mobility | 22.99 ± 4.90 | 20.09 ± 5.23 | 17.45 ± 4.75 | –0.093 | < 0.001 |
| Self-care | 22.58 ± 5.04 | 19.40 ± 5.30 | 18.14 ± 4.88 | –0.063 | < 0.001 |
| Usual activities | 22.72 ± 5.01 | 19.86 ± 5.22 | 18.49 ± 4.21 | –0.076 | < 0.001 |
| Pain or discomfort | 22.94 ± 4.97 | 21.53 ± 5.11 | 18.55 ± 4.97 | –0.097 | < 0.001 |
| Anxiety or depression | 22.62 ± 5.06 | 21.33 ± 5.10 | 19.15 ± 5.45 | –0.065 | < 0.001 |

Abbreviations: EQ-5D: European Quality of Life Scale-Five Dimensions.

^￡^Level ‘1’: no problems, ‘2’: some problems, ‘3’: extreme problems.

*P-value was adjusted by age and pulmonary function (normal, restrictive, and obstructive).

**e-Table 2. Association with handgrip strength and Health-related Quality of Life Instrument with 8 Items (n = 3723)**

1. Association with handgrip strength and HINT-8 in male participants (n = 1597)

| Level^￡^ | 1 | 2 | 3 | 4 | Beta | P-value* |
| --- | --- | --- | --- | --- | --- | --- |
| Climbing stairs | 38.53 ± 7.34 | 35.57 ± 7.83 | 30.33 ± 8.86 | 36.51 ± 7.05 | –0.095 | < 0.001 |
| Pain | 37.90 ± 7.47 | 36.67 ± 8.04 | 34.55 ± 9.21 | 30.32 ± 7.08 | –0.088 | 0.002 |
| Vitality | 37.50 ± 7.67 | 38.30 ± 7.50 | 36.05 ± 7.81 | 30.07 ± 8.85 | –0.167 | < 0.001 |
| Working | 38.35 ± 7.62 | 36.07 ± 7.41 | 32.48 ± 8.93 | 29.53 ± 8.44 | –0.177 | < 0.001 |
| Depression | 37.56 ± 7.76 | 36.65 ± 7.77 | 34.97 ± 8.00 | 31.7 ± 12.47 | –0.136 | < 0.001 |
| Memory | 37.97 ± 7.47 | 36.78 ± 7.89 | 32.91 ± 9.20 | 23.92 ± 11.02 | –0.034 | 0.226 |
| Sleep | 37.62 ± 7.73 | 37.08 ± 7.67 | 34.44 ± 9.32 | 31.30 ± 6.20 | –0.082 | 0.004 |
| Happiness | 37.26 ± 8.30 | 38.33 ± 6.90 | 36.39 ± 7.80 | 33.30 ± 10.27 | –0.088 | 0.002 |

^￡^Level ‘1’: no problems, level ‘2’: mild, level ‘3’: moderate, level ‘4’: severe problems.

*P-value was adjusted by age and pulmonary function (normal, restrictive, and obstructive).

1. Association with handgrip strength and HNT-8 in female participants (n=2126)

| Level^￡^ | 1 | 2 | 3 | 4 | Beta | P-value* |
| --- | --- | --- | --- | --- | --- | --- |
| Climbing stairs | 22.96 ± 4.72 | 21.45 ± 4.69 | 20.00 ± 5.18 | 18.82 ± 5.26 | –0.107 | < 0.001 |
| Pain | 22.50 ± 4.71 | 22.08 ± 4.85 | 19.96 ± 5.18 | 19.28 ± 4.18 | –0.078 | 0.001 |
| Vitality | 22.66 ± 4.73 | 22.75 ± 4.67 | 21.31 ± 4.83 | 18.76 ± 5.01 | –0.142 | < 0.001 |
| Working | 22.69 ± 4.75 | 21.80 ± 4.72 | 19.37 ± 4.99 | 18.18 ± 5.01 | –0.116 | < 0.001 |
| Depression | 22.06 ± 4.78 | 22.07 ± 4.92 | 21.02 ± 5.41 | 18.71 ± 4.63 | –0.035 | 0.128 |
| Memory | 22.29 ± 4.74 | 21.93 ± 4.92 | 19.89 ± 5.02 | 18.78 ± 6.02 | –0.001 | 0.950 |
| Sleep | 22.13 ± 4.87 | 22.17 ± 4.81 | 20.82 ± 5.11 | 19.76 ± 3.96 | 0.003 | 0.910 |
| Happiness | 21.53 ± 4.71 | 22.90 ± 4.88 | 21.79 ± 4.82 | 19.75 ± 4.80 | –0.038 | 0.096 |

^￡^Level ‘1’: no problems, level ‘2’: mild, level ‘3’: moderate, level ‘4’: severe problems.

*P-value was adjusted by age and pulmonary function (normal, restrictive, and obstructive).
